# The Evolution of the Modern Hospital

**Published:** 1896-08-22

**Authors:** 


### The Evolution of the Modern Hospital.

### III.—ITS BENEFITS AND LESSONS.

The Brook Hospital described last week reminds us in a degree of the hospital at Pullmantown. It is a small town in itself as, in addition to the 506 beds which the hospital may ultimately contain, the staff numbers no less than 325 persons. The medical superintendent is provided with a separate house to himself. The nurses are provided with a home consisting of three blocks of buildings under the control of the matron, the charge nurses occupying the main block where the dining and general sitting rooms are placed, the day assistant nurses another block, and lastly—a most excellent arrangement—the night nurses, eighty in number, have one whole block entirely given up to them. The female servants have a second home under the control of the housekeeper, and the male servants occupy a third home under the supervision of the steward. These two great ideas of disconnecting the houses occupied by the staff from the infected area, and the placing of the members of each division of the staff altogether, but in separate buildings, under their respective heads, are features highly to be commended, fraught with important bearings upon the well-being and discipline of the whole establishment, whilst they provide an object lesson for all who have to do with establishments where a great number of people are constantly employed.

The Brook Hospital is the first of three great hospitals now in course of construction under the direction of the Metropolitan Asylums Board. There can be no question that it is a great conception, marking a new departure and a distinct advance in our social system, for there has never before been any modern hospital devoted to infectious diseases of anything like the size and completeness of the Brook Hospital in the history of the world. Taken as a whole, it is greatly to the credit of all who have had any part in its conception and completion that it should represent the evolution of hospital development in this country and abroad. As a mere mass of buildings devoted entirely to the reception of sick people suffering from acute disease of an infectious character and to their attendants, it is full of interest and instruction, and may be regarded as an apt illustration of the vast outlay entailed and of the results achieved by our modern civilisation. The expenditure alone, including the site and furniture, already amounts to about £280,000, or nearly £600 per bed on the 488 beds it contains. Such an expenditure is a remarkable fact, especially when we recall the hostile criticism to which the Governors of St. Thomas's Hospital had to submit at the time of its erection and since, seeing that the cost per bed at St. Thomas's Hospital was £692 on a total of 573 beds, or about £770 if the £85 per bed attributable to the foundations be added. Taken as a whole, the Brook Hospital is undoubtedly an object lesson for all classes. It shows a breadth of view and a comprehension of much which goes to the production of a perfect hospital, which is highly commendable, and cannot fail to cause both criticism and commendation. To whichever aspect the reader inclines, we are confident that no one with eyes to see and a mind open to instruction who takes an opportunity of visiting the Brook Hospital, or the Great Northern Central Hospital, Holloway, and studying them as a whole, can fail to come away benefited, because his view must have been extended and his comprehension of the trend of public opinion and public demands upon local authorities, Parliament, and the managers of the voluntary hospitals, must have immeasurably increased.

We have ventured to suggest a visit to the Great Northern Central Hospital, because that institution is a remarkable example of the evolution of the hospital idea. It was erected on its present site nine years ago, in response to a demand for a great general hospital which the inhabitants of North London made on their own behalf, and which they have nobly gratified at their own expense. It is the only hospital in London which combines the rectangular and circular system of construction carried out upon an identical scale, so that the relative advantages or defects of either system can be accurately ascertained and demonstrated by the practical men who devote attention to such matters. It is the only hospital in this country which has been planned to provide accommodation in large wards for the poorer classes of hospital patients, and which has, at the same time, a separate wing constructed entirely for the accommodation of patients who can afford to pay, at any rate something, for treatment during their stay in the hospital. It follows, therefore, that it is the only hospital which we have where nurses can be completely trained, because a nurse at the Great Northern Central Hospital not only learns all the duties appertaining to the care and succour of the sick in general wards, but she is enabled to be trained in the attendance upon private patients, so that her value as a nurse is immeasurably increased should she determine to attach herself to an institution which supplies nurses to the public. The Great Northern Central Hospital has, further, an out-patient department, which is, on the whole, probably the completest and simplest from the administrator's and architect's point of view. The out-patient department is further conducted upon principles which commend themselves to the approval not only of the subscribers, but of all classes of the medical profession. On a recent occasion we took one of the greatest authorities on hospitals in the world to make an inspection of the Great Northern Central Hospital, and his statement was—and he had seen many hospitals in many countries—that he considered it one which no person should fail to visit who took an interest in hospitals, or who had any idea of erecting a new one. Finally, this hospital is supported as no other London hospital is supported, because the revenues are supplied by the people of the district from which the patients are mainly drawn.

There can be no doubt that the more the average man and woman is induced to consider the modern hospital from the standpoint of its value and necessity to society and to all classes, the better must be the quality of the work done, and the greater the volume of lessened suffering throughout the world of to-day. A hospital has long ceased in public estimation to be a building to be placed out of sight, and to be avoided by the hale of all classes. It is rapidly assuming its proper place in public estimation as an institution with which the most vital interests of all classes are most closely identified. But unless it is maintained in the maximum of efficiency, not only the masses of the population, but every member of every class will immeasurably suffer from the circumstance that the modern hospital has been allowed to secure less than its fair share of public attention and public support.

